# *GALC* Deletions Increase the Risk of Primary Open-Angle Glaucoma: The Role of Mendelian Variants in Complex Disease

**DOI:** 10.1371/journal.pone.0027134

**Published:** 2011-11-04

**Authors:** Yutao Liu, Jason Gibson, Joshua Wheeler, Lydia Coulter Kwee, Cecile M. Santiago-Turla, Stephen K. Akafo, Paul R. Lichter, Douglas E. Gaasterland, Sayoko E. Moroi, Pratap Challa, Leon W. Herndon, Christopher A. Girkin, Donald L. Budenz, Julia E. Richards, R. Rand Allingham, Michael A. Hauser

**Affiliations:** 1 Center for Human Genetics, Duke University Medical Center, Durham, North Carolina, United States of America; 2 Department of Medicine, Duke University Medical Center, Durham, North Carolina, United States of America; 3 Duke University Eye Center, Duke University Medical Center, Durham, North Carolina, United States of America; 4 Unit of Ophthalmology, Department of Surgery, University of Ghana Medical School, Korle Bu, Ghana; 5 Department of Ophthalmology and Visual Sciences, University of Michigan, Ann Arbor, Michigan, United States of America; 6 Department of Epidemiology, University of Michigan, Ann Arbor, Michigan, United States of America; 7 Eye Doctors of Washington, Chevy Chase, Maryland, United States of America; 8 Department of Ophthalmology, University of Alabama at Birmingham, Birmingham, Alabama, United States of America; 9 Department of Ophthalmology, Bascom Palmer Eye Institute, Miami, Florida, United States of America; Institut Jacques Monod, France

## Abstract

DNA copy number variants (CNVs) have been reported in many human diseases including autism and schizophrenia. Primary Open Angle Glaucoma (POAG) is a complex adult-onset disorder characterized by progressive optic neuropathy and vision loss. Previous studies have identified rare CNVs in POAG; however, their low frequencies prevented formal association testing. We present here the association between POAG risk and a heterozygous deletion in the galactosylceramidase gene (*GALC*). This CNV was initially identified in a dataset containing 71 Caucasian POAG cases and 478 ethnically matched controls obtained from dbGAP (study accession phs000126.v1.p1.) (p = 0.017, fisher's exact test). It was validated with array comparative genomic hybridization (arrayCGH) and realtime PCR, and replicated in an independent POAG dataset containing 959 cases and 1852 controls (p = 0.021, OR (odds ratio) = 3.5, 95% CI −1.1–12.0). Evidence for association was strengthened when the discovery and replication datasets were combined (p = 0.002; OR = 5.0, 95% CI 1.6–16.4). Several deletions with different endpoints were identified by array CGH of POAG patients. Homozygous deletions that eliminate GALC enzymatic activity cause Krabbe disease, a recessive Mendelian disorder of childhood displaying bilateral optic neuropathy and vision loss. Our findings suggest that heterozygous deletions that reduce GALC activity are a novel mechanism increasing risk of POAG. This is the first report of a statistically-significant association of a CNV with POAG risk, contributing to a growing body of evidence that CNVs play an important role in complex, inherited disorders. Our findings suggest an attractive biomarker and potential therapeutic target for patients with this form of POAG.

## Introduction

Glaucoma is the leading cause of irreversible blindness worldwide [1]. As a heterogeneous group of disorders, glaucoma is a pathologic condition characterized by progressive loss of retinal ganglion cells with corresponding loss of the visual field. Primary open-angle glaucoma (POAG, OMIM #137760) is the most common form of glaucoma [2]. POAG is characterized by typical glaucomatous changes of the optic nerve and vision loss in the absence of secondary causes. Well recognized risk factors for the development of POAG include elevated intraocular pressure (IOP), increasing age, African ancestry, and family history of glaucoma [3], [4]. Genetics plays an important role in the pathogenesis of POAG [2], [3], [5]. Traditional linkage studies have identified many genomic regions (GLC1 loci) for familial cases [6]. Among these, mutations in several genes, such as myocilin, optineurin, *WDR36* (WD repeat domain 36) and *CYP1B1* (cytochrome P450 1B1), are causal or associated with increased risk of developing POAG in multiple populations [7], [8], [9], [10], [11]. Association studies have identified many variants that may contribute to POAG [6], [12]. Recently, genome-wide association studies have been used to identify genetic risk factors for POAG or glaucoma-related ocular phenotypes [13], [14], [15], [16], [17], [18], [19]. These genes and regions include caveolin 1 and 2 (*CAV1/2*), transmembrane and coiled-coil domains 1 (*TMCO1*), SIX homeobox 1 (*SIX1*), and cyclin-dependent kinase inhibitor 2B (*CDKN2B*) [13], [19], [20].

In addition to DNA sequence variation, DNA copy number variations (CNV) have been increasingly reported in human genetic disorders such as autism, HIV/AIDS, and cancer [21]. However, the role of CNV in POAG has been unclear. Abu-Amero et al. found no evidence of CNVs associated with POAG among 27 POAG cases and 12 controls using array comparative genomic hybridization (array CGH) [22]. However, in a larger dataset, Davis et al. using SNP genotyping arrays [23] studied 400 POAG cases and 500 non-glaucoma controls and found eleven rare CNVs that may be relevant to POAG. Fingert et al. recently identified a large duplication of *TBK1* (Tank-binding kinase 1) in patients with normal tension glaucoma [24]. Replications of these studies in larger datasets will help to clarify the importance of these CNVs in glaucoma risk.

In order to systematically examine the role of CNVs in POAG, we performed a discovery project using a genome-wide SNP genotyping array in a POAG case-control dataset. A set of candidate CNVs was selected for validation using realtime PCR. One specific CNV in the galactosylceramidase (*GALC*, OMIM *606890) gene was further examined in an independent case-control dataset. The heterozygous loss of the *GALC* gene was confirmed using three-primer PCR assays and array CGH.

## Results

In the discovery phase of our study, we performed genome-wide DNA copy number variant analysis in 92 POAG cases genotyped on the Illumina HumanHap610 BeadChip. The CNV frequencies in our POAG patients were compared to publicly available controls genotyped on the Illumina HumanCNV370 BeadChip (dbGAP study accession phs000126.v1.p1.c1). The analysis was limited to the 363,185 markers shared between the two BeadChip platforms. After applying quality control protocols described in the methods section, 71 POAG samples and 478 dbGAP controls (greater than 40 years old) were analyzed using PennCNV software. Overall, 15,940 CNV calls were generated for a total of 549 DNA samples, with an average of 29 CNV events per sample. About one third of CNV calls overlapped with known coding genes. We performed a case-control comparison with all the identified CNVs. However, none of the CNVs reached genome-wide significance (p value<5×10^−8^). CNVs in five genomic regions were selected for further follow-up based on their potential functional annotation and relative frequency in cases versus controls. These five candidate regions were *RASA4* (RAS p21 protein activator 4), *EYA1* (eyes absent 1 homolog 1), *CROCC* (ciliary rootlet coiled-coil, rootlletin), *ALDH1A2* (aldehyde dehydrogenase 1 family, member A2), and *GALC* (galactosylceramidase).

**Figure 1:**
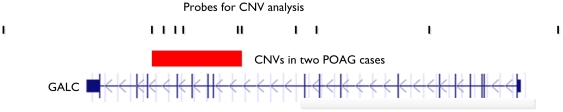
Heterozygous DNA deletion in the GALC gene. A heterozygous DNA deletion in the galactosylceramidase (*GALC*) gene present in 2 of 71 POAG cases and absent in 478 controls. The deletion was identified through six probes common to both the Illumina HumanCNV370-Duo and Human610-Quad BeadChips. The deletion indicated by the red-colored box includes five exons (exons 11–15) and abolishes enzymatic activity.

**Figure 2:**
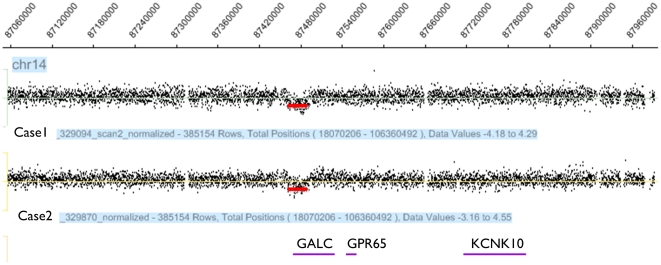
Validation of *GALC* deletion with array CGH. The *GALC* deletion from Figure 1 was validated with high resolution chromosome 14 specific CGH array. This figure showed a 31 kb deletion in the *GALC* gene, indicated by the red line.

In order to validate the CNV calls generated from the SNP genotyping array, we used TaqMan-based realtime PCR on the 71 genotyped POAG cases using probes in these five selected regions. Realtime PCR validated the presence of the *GALC* deletion, but not the CNV calls in the regions of *RASA4*, *EYA1*, *CROCC*, or *ALDH1A2*. In the discovery dataset, 2/71 (2.8%) POAG cases were identified with heterozygous deletions in *GALC* while none were found in 478 age-matched US Caucasian controls (**Figure 1**). The difference in allele frequency between cases and controls was statistically significant (p = 0.017, Fisher's exact test). The *GALC* deletion was further validated in the POAG cases using a human chromosome 14-specific CGH array (Roche NimbleGen, Inc., Madison, WI) which demonstrated that it encompasses a 31 kb genomic region (**Figure 2**).

Association of the *GALC* CNV was replicated in a second Caucasian POAG case/control dataset. This dataset consisted of 959 cases and 1104 examined normal controls genotyped with realtime PCR that were augmented by an additional 748 publicly available population controls (greater than 40 years old) genotyped with the Affymetrix 6.0 array [25]. In this analysis, 9/959 POAG cases (0.94%) and 5/1852 controls (0.27%) carried the heterozygous DNA loss in *GALC* (P = 0.021, Fisher's exact test; OR = 3.5, 95%CI 1.1–12.0). After combining the discovery and replication datasets the strength of association was increased (p = 0.002, Fisher's exact test, OR = 5.0, 95% CI 1.6–16.4). The CNV deletion for *GALC* was found in 1.07% of POAG cases and 0.21% of the controls in the combined Caucasian dataset. There were no significant differences in age-at-diagnosis, maximum IOP, or visual acuity in POAG cases with the *GALC* deletion compared with POAG cases without the deletion. Realtime PCR-based screening was applied to African American (400 cases/290 controls) and West African (Ghana) (190 cases/500 controls) case-control datasets. No *GALC* CNV deletions were identified in either dataset (**Table 1**). This suggests that *GALC* deletions are either absent or extremely rare in individuals of West African ancestry. This finding is supported Shaikh *et al*. who report that *GALC* deletions were absent in 694 African American samples [25].

**Table 1:** Summary of *GALC* deletion screening in all datasets.

Dataset	Ethnicity	Sample size(cases/controls)	Deletion events(cases/controls)	Fisher's exact p-value	Odds Ratio (95% CI)	Experimental Platform
Discovery	Caucasian	71/478	2 (2.8%)/0	0.017	Infinite	SNP-based array
Replication	Caucasian	959/1852	9 (0.94%)/5 (0.27%)	0.021	3.5 (1.1–12.0)	Realtime PCR+SNP-based array
Combined	Caucasian	1030/2330	11 (1.07%)/5 (0.21%)	0.002	5.0 (1.6–16.4)	Array & PCR
Duke	African American	400/290	0/0	1.000	N/A	Real time PCR
CHOP	African American	0/694	0/0	1.0	N/A	SNP-based array
Duke	Ghanaian	190/500	0/0	1.000	N/A	Real time PCR

The CHOP dataset was obtained from the Copy Number Variation project at the Children's Hospital of Philadephia (CHOP) [25]; Fisher's exact p-value was based on one-tailed test, as we hypothesize that loss or decrease of GALC function results in POAG risk.

**Figure 3:**
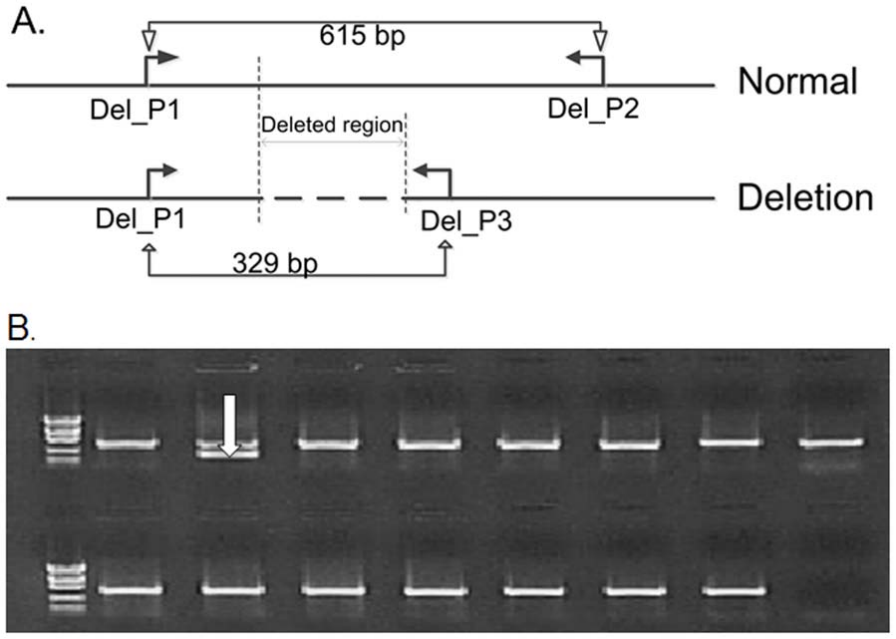
Three-Primer PCR assay for *GALC* deletion. (A) Schematic diagram of a portion of the *GALC* gene showing the region around the known GALC deletion. Both primers Del_P1 and Del_P3 are located outside of the deletion while primer Del_P2 is inside the deletion and close to the left breakpoint. (B) DNA samples with heterozygous deletion produced two DNA bands (329 bp and 615 bp in size) while DNA samples without GALC deletion generated one DNA band of 615 bp in size. The white arrow indicates the DNA sample with heterozygous deletion.

**Figure 4:**
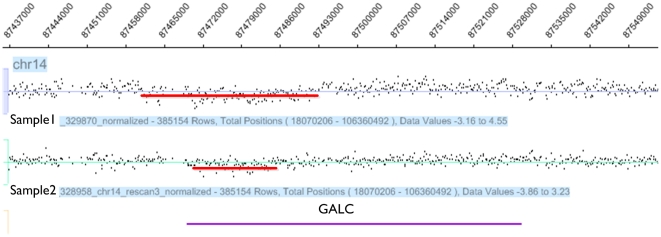
Heterogeneity of *GALC* deletions in different DNA samples. Two heterozygous *GALC* deletions with different end-points were identified by array CGH, indicated by the red line. The lower diagram shows the previously reported 31 kb *GALC* deletion while the upper diagram shows a smaller deletion of approximately 15 kb.

To further define the observed deletion, we used a 3-primer PCR assay (**Figure 3**) to examine our POAG cases and controls [26], [27], [28]. Three samples carry a previously reported 31 kb deletion (Human genome build 37, chromosome 14: 88,391,505–88,423,176) while the others have deletions with previously unreported and variable endpoints in our POAG cases. The heterogeneity of *GALC* CNV events was validated by array CGH (**Figure 4**). The 31 kb *GALC* deletion has been reported to carry a 4 bp direct repeat (TATC) at the deletion junction [26].

## Discussion

We have identified CNV deletions of *GALC* that, when heterozygous, increase risk of POAG by four-fold in Caucasian individuals. CNV deletions were not found in subjects of African ancestry. The normal *GALC* mRNA encodes an 80 kDa precursor, which is processed into 50 and 30 kDa subunits to form an enzymatically active complex [26], [29]. The 31 Kb *GALC* deletion abolishes production of the 30 kDa subunit and results in the production of a significantly shortened (15%) 50 kDa subunit [26]. *GALC* encodes the enzyme galactosylceramidase, which is required for the degradation of specific galactolipids in the white matter of the central and peripheral nervous system. These galactolipids include galactosylceramide, galactosylsphingosine (psychosine), lactosylceramide, and monogalactosyldiglyceride. GALC is critical in the maintenance of the myelin sheath, which functions as the protective covering around certain nerve cells to ensure the rapid transmission of nerve impulses. Psychosine forms during the production of myelin and is catabolized in part by GALC. Normally, only small amounts of psychosine are present in the brains of individuals with normal GALC enzymatic activity; however complete loss of activity leads to accumulation of psychosine and galactosylceramide, resulting in the development of Krabbe disease, a form of globoid cell leukodystrophy. Krabbe disease is a rare, autosomal recessive disorder of the central and peripheral nervous system [30] that is characterized by increased muscle tone, impaired motor control, seizures, hearing loss and vision loss.

Among all the *GALC* mutations causing Krabbe disease, the most common in Caucasian individuals (40–45%) is the large 31-kb deletion beginning in intron 10 and continuing past the end of *GALC* [29], [30]. This deletion has not been reported in African American or African Krabbe disease patients. Based on the incidence of Krabbe disease in the United States (1 in 100,000 births) [29], [30], the estimated combined frequency of all functionally-relevant *GALC* mutations is 0.003, implying a population frequency of the 30-kb deletion of approximately 0.0013, very similar to the frequency of 0.0011 we have detected in the present study (5 deletions in 4660 chromosomes of 2330 controls). This frequency match suggests that the controls included in our studies are appropriate and representative of the overall Caucasian population.

Although optic neuropathy with associated loss of vision is one of the most common clinical manifestations of Krabbe disease, poor patient prognosis has limited studies of the molecular mechanism of vision loss. In 1978, Brownstein et al. reported optic atrophy in an infant Krabbe disease patient [31]. Absence of GALC activity and accumulation of its substrates, galactosylcerebroside and psychosine, in the optic nerve caused optic nerve fiber and ganglion cell layer degeneration [31]. The carriers of *GALC* mutations are known to have reduced enzymatic activity [29], [30], and we hypothesize that this reduced activity is the mechanism by which *GALC* deletions increase risk of POAG. Measurements of GALC enzymatic activity could be used to screen patients for risk of POAG. Currently it is estimated that there are hundreds of thousands of individuals who carry *GALC* deletions in the United States [29], [30]. The findings reported here show that these carriers are at increased risk for glaucoma. In addition, the variability of GALC activity in the general population is broad. These estimates suggest that a substantial fraction of the population is expected to have as little as 20–30% of the normal GALC enzymatic activity levels [29], [30]. It will be important to examine POAG-related phenotypes in the parents or grandparents of Krabbe disease patients, especially those GALC deletion carriers. This examination will provide direct evidence to the functional involvement of GALC in the pathogenesis of glaucoma. This work is currently underway.

McCarroll et al. and Redon et al. have reported two CEPH individuals (NA12716 and NA11840) with a heterozygous 24 kb deletion in the *GALC* gene using the Affymetrix 6.0 array [32], [33]. Supplementary data from two genome-wide expression studies using HapMap samples [34], [35], indicates that the *GALC* gene expression in these two CEPH individuals is the lowest in their respective families (pedigrees 1358 and 1349). Further, the GALC activity for these individuals is in the lowest 20th percentile compared to 90 other Caucasian subjects. These data suggest that GALC enzymatic activity may constitute a potential biomarker as well as therapeutic target for POAG.

In conclusion, this is the first study to report the role of heterozygous *GALC* deletions as a significant risk factor for POAG. We hypothesize that increased POAG risk is mediated through reduced gene expression and enzymatic activity that in turn leads to functional deficits in retinal ganglion cells. Further studies are warranted on the role of GALC in optic nerve function, the impact of *GALC* mutations in pathogenesis of glaucoma, and galactosylceramidase activity as a biomarker and a potential therapeutic target for POAG.

## Materials and Methods

### Ethics Statement

This study was reviewed and approved by the Institutional Review Board of Duke University Medical Center (Durham, NC) and adhered to the tenets of the Declaration of Helsinki. Written informed consent was obtained from all study participants.

### Study Sample and Phenotype Description

Subjects with POAG were unrelated and met the following inclusion criteria [36] : 1) glaucomatous optic neuropathy in both eyes; 2) visual field loss consistent with optic nerve damage in at least one eye. Glaucomatous optic neuropathy was defined as a cup-to-disc ratio greater than 0.7 or focal loss of the nerve fiber layer resulting in a notch in the neuroretinal rim, associated with a glaucomatous visual field defect. Visual fields were performed using standard automated perimetry or frequency doubling test (FDT) [1]. IOP was not used as an inclusion criterion. The exclusion criteria for POAG subjects included the diagnosis or history of a secondary form of glaucoma or history of ocular trauma. Medical records for all POAG cases and control subjects were reviewed by professionally trained glaucoma subspecialists (RRA, SEM). The examined control subjects were unrelated and met the following criteria: 1) no self-report of a first-degree relative with glaucoma; 2) IOP less than 21 mmHg in both eyes without treatment; 3) no evidence of glaucomatous optic neuropathy in either eye; 4) normal visual field in both eyes.

We have used a two–stage study design: discovery and replication phases. The Caucasian POAG cases in the discovery phase were recruited at Duke University Eye Center. The Caucasian controls in the discovery phase were controls from a previous Parkinson's GWAS study (dbGAP study accession phs000126.v1.p1.c1). This study only uses those controls at least 40 years of age, with a total of 478 individuals. These controls are negative for neurologic disease and do not have a family history of any neurodegenerative disease. They are Caucasian and non-Hispanic subjects. They were enrolled at either Boston University or Indiana University and deposited into the NINDS Repository. The Caucasian POAG cases in the replication phase were enrolled at several sites: Duke University Eye Center, the University of Michigan Kellogg Eye Center, or Eye Doctors of Washington at Chevy Chase, MD. The examined control individuals in the replication phase were enrolled at either Duke University Eye Center or the University of Michigan Kellogg Eye Center. In the replication phase, we also included 748 US Caucasian controls from a previous Bipolar dbGAP GWAS study (phs000017.v2.p1) with an age cut-off at 40 years old. These controls will serve as non-examined population controls. These controls were genotyped using Affymetrix 6.0 array containing 1,800,000 probes for CNV analysis. All the African American POAG cases and controls were enrolled at Duke University Eye Center. The Ghanaian POAG cases and controls were collected in Ghana (West Africa).

### CNV Calling

Extraction of genomic DNA by standard techniques (Gentra, Minneapolis, MN) was carried out for 1130 Caucasian POAG cases, 1350 Caucasian controls, 400 African American POAG cases, 300 African American controls, 190 Ghanaian POAG cases and 500 Ghanaian controls. Ninety-two POAG cases were subjected to genome-wide SNP genotyping with the Infinium II Human610-Quad BeadChip (Illumina, San Diego, CA, USA) at the Molecular Genetics Core of the Center for Human Genetics at Duke University. High quality genomic DNA (500 ng) was used to genotype each sample according to the manufacturer's guidelines [37]. BeadStudio software (Illumina) was used to analyze DNA genotype and intensity. For 943 controls we obtained the genotype and intensity files from dbGAP (study accession phs000126.v1.p1). These samples were genotyped using Illumina HumanCNV370-Duo BeadChip. For CNV analysis, samples were required to have the following criteria: First, only samples with call rates >98% were included. Second, only samples with the standard deviation of normalized intensity (Log R Ratio, LRR) ≤0.35 were included. Third, only samples where the correlation of LRR to wave model ranged between −0.2 and 0.4 were included. Finally, only samples with the initial CNV call count ≤40 were included in the analysis. A total of 71 POAG cases and 478 controls met all inclusion measures in the final CNV analysis. For those 748 GAIN controls genotyped with Affymetrix 6.0 array, we applied a similar quality control procedure: 1) standard deviation of LRR ≤0.35; 2) a minimum of 5 probes per CNV due to a much higher density of probes (a total of 1.8 million probes per sample); 3) ≤100 CNV calls per sample; 4) drift of B allele frequency (BAF) ≤0.01; and 5) wave factor ≤0.05.

To call CNVs, we used the PennCNV algorithm [38], which combines multiple sources of information, including LRR and BAF at each SNP marker. We required a minimum of 3 consecutive probes for a CNV call. Since POAG cases and controls were genotyped with different types of Illumina Beadchips, only SNPs and probes shared in common between these two Beadchips (approximately 370,000) were utilized to generate CNV calls. The differences in CNV frequency between POAG cases and controls were evaluated using Fisher's exact test. A list of specific CNVs was selected for further validation using realtime PCR in a large POAG case/control dataset.

### CNV Validation and Screening by Realtime PCR

To validate the CNVs determined by PennCNV, we selected five candidate CNVs identified in the initial genome-wide screen. We used TaqMan® Copy Number Assays from ABI (Applied Biosystems Inc, Carlsbad, CA, USA) on the ABI Prism® 7900HT Sequence Detection System for validation and further screening in additional POAG cases and controls. A VIC-labeled Copy Number Assay for RNase P was selected to be an endogenous control as it performed in the same reaction with gene-specific assays. Only one probe was selected for each CNV region. Each sample was assayed with four replicates by using 10 ng DNA in each reaction in 384-well format. The CNV calls were generated with SDS software and CopyCaller™ from ABI (Applied Biosystems Inc, Carlsbad, CA, USA). A known CEPH sample was used as a reference for a copy number of 2. This CEPH sample was already examined by SNP microarray to contain two copies of candidate CNV regions. In order to make CNV calls in CopyCaller™ software, a confidence score of greater than 0.95 was required with four replicates.

The CNV assay for *EYA1* gene used Hs03668790 to target intron 7. The assay for the *CROCC* gene used Hs01817683 to target the boundary of exon 3 and intron 3. The assay for *ALDH1A2* gene used Hs02281830 to target the boundary of intron 7 and exon 8. The assay for *RASA4* was custom-designed to target the genomic region between exon 1 and exon 3. The specific CNV assay for the *GALC* gene used Hs00121467 to target exon 13. The assay for *GALC* was run with all Caucasian, African American, and Ghana case-control samples as well as a multiplex familial POAG cohort.

### Three-primer PCR assay for *GALC* Deletion

We utilized a three-primer PCR assay that was developed previously [26] to examine a known deletion in the *GALC* gene. The primer sequences were Del_P1: 5′-CCTATATGGAAAACAATGTGG-3′, Del_P2: 5′-AAGGAGCTAACATTTCAGGC-3′; and Del_P3: 5′- AAGGAGCTAACATTTCAGGC-3′. Primers Del_P1 and Del_P3 were outside of the genomic deletion, while primer Del_P2 was inside of the deleted region and close to Del_P1 (**Figure 3**). Individuals with a homozygous deletion generate a 329 bp fragment with PCR primers Del_P1 and Del_P3 while subjects without deletion produce a 615 bp fragment with PCR primers Del_P1 and Del_P2. Individuals with a heterozygous deletion generate two PCR fragments of 329 and 615 bp in size. This three-primer PCR reaction was used to examine all POAG cases and controls.

### Validation with Array CGH

Selected DNA samples were examined with CGH array experiments for CNV changes in *GALC*. A high-resolution chromosome 14 specific CGH array was used according to the standard protocols provided by the manufacturer (Roche-NimbleGen, Inc, Madison, WI, USA) [39]. For each sample, 1 µg high quality undegraded DNA was included with human reference DNA from HapMap. The human chromosome 14-specific CGH array provides measurements from 385,000 unique genomic loci on chromosome 14. The experimental sample was labeled with Cy3 and reference sample was with Cy5. The arrays were scanned on an Axon 4100A scanner (Molecular Devices, Sunnyvale, CA, USA). TIFF images were analyzed using NimbleScan 2.5 software to obtain fluorescent intensity and the copy number was further determined using the segMNT algorithm with a 10× averaging window and minimum difference in log ratio of 0.2. A minimum of 10 consecutive probes was required to make CNV calls. The copy number variation result was visualized using SignalMap 1.8 from Roche NimbleGen.
