# Correction: *GALC* Deletions Increase the Risk of Primary Open-Angle Glaucoma: The Role of Mendelian Variants in Complex Disease

**DOI:** 10.1371/journal.pone.0103197

**Published:** 2014-07-14

**Authors:** The PLOS ONE Staff

There is an error in one of the primer sequences listed in the Materials and Methods section. In the second sentence under the subheading “Three-primer PCR assay for *GALC* Deletion”, the correct primer sequence for primer sequence Del_P3 is 5’-TCAAGTCCTTGATGATCACC-3’.
